# Past, Present, and Future of RNA Modifications in Infectious Disease Research

**DOI:** 10.1021/acsinfecdis.4c00598

**Published:** 2024-11-21

**Authors:** Xiaoqing Pan, Alexander Bruch, Matthew G. Blango

**Affiliations:** Junior Research Group RNA Biology of Fungal Infections, Leibniz Institute for Natural Product Research and Infection Biology: Hans Knöll Institute (HKI), 07745 Jena, Germany

**Keywords:** RNA modification, RNA editing, m^6^A, fungi, bacteria, virus

## Abstract

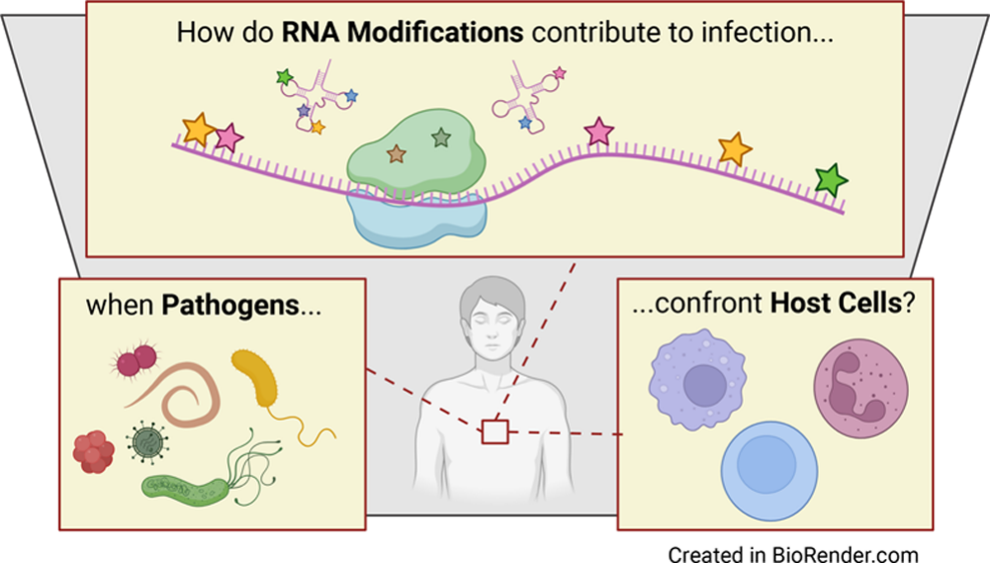

In early 2024, the National Academies of Sciences, Engineering, and Medicine (NASEM) released a roadmap for the future of research into mapping ribonucleic acid (RNA) modifications, which underscored the importance of better defining these diverse chemical changes to the RNA macromolecule. As nearly all mature RNA molecules harbor some form of modification, we must understand RNA modifications to fully appreciate the functionality of RNA. The NASEM report calls for massive mobilization of resources and investment akin to the transformative Human Genome Project of the early 1990s. Like the Human Genome Project, a concerted effort in improving our ability to assess every single modification on every single RNA molecule in an organism will change the way we approach biological questions, accelerate technological advance, and improve our understanding of the molecular world. Consequently, we are also at the start of a revolution in defining the impact of RNA modifications in the context of host–microbe and even microbe–microbe interactions. In this perspective, we briefly introduce RNA modifications to the infection biologist, highlight key aspects of the NASEM report and exciting examples of RNA modifications contributing to host and pathogen biology, and finally postulate where infectious disease research may benefit from this exciting new endeavor in globally mapping RNA modifications.

## NASEM Report as a Framework for the Advancement of RNA Modifications Research

The new report from the National Academies of Sciences, Engineering, and Medicine (NASEM) entitled “Charting a future for sequencing RNA and its modifications: A new era for biology and medicine” issues a blueprint for the future of RNA and RNA modification research.^1^ The report, coordinated and assembled by a diverse list of international leaders, first provides a historical overview of RNA modification research and its vast impact on our understanding of biology and disease. The authors then go to great lengths to explain the cutting-edge of technologies for mapping RNA modifications and where we are likely to see rapid advances in the near and long-term. In defining the required drivers of acceleration for RNA modifications research, the authors identify the importance of not only technological advances, but also improved chemical standards and computational solutions, e.g., databases, in facilitating this revolution in our understanding of RNA. In addition to the need for advances in technology and reagents, including novel modalities for modification detection, improved biochemical standards, and overhauled or novel computational approaches, the report finds that we will also require buildup of infrastructure, training of a skilled workforce, and increased public awareness in parallel. The authors end with a vision for the future of RNA modification research, including a series of succinct recommendations for the community and society at large moving forward. The document is expansive and inspirational in its scope for the future of RNA research, covering everything from the basic biology and biochemistry of RNA modifications to a concrete plan for coordination and allocation of resources and effort. It will certainly serve as a tool for education, a guide for policy makers, and a roadmap for future RNA researchers. Collectively, the NASEM report is a call to arms, encouraging further investment in RNA and RNA modification research and global cooperation in improving our understanding of these crucial chemical changes.

Throughout the report, the impact of RNA modifications on disease and infection is touched upon, but as expected from such an expansive report, a full dissection of the field is impossible. Here, we aim to add to the importance of this seminal report with additional insights into where the field of RNA modification research intersects that of infection biology. We provide a more elaborate description of key references and posit just a few ways that the success of the proposed path from the NASEM report could change the way we consider infectious diseases. We encourage the readers of this perspective to also visit the NASEM report to better appreciate the entire landscape of RNA modifications research, through the lens of many of the very researchers that made seminal discoveries to establish the field.

## What Is an RNA Modification and How Do We Map Them?

RNA is decorated with over 170 known chemical modifications that influence RNA function, stability, structure, and interaction with other macromolecules including nucleic acids and proteins. These chemical modifications range from small alterations like methylations and acetylations to bulky modifications like those of glutamyl-queuosine (GluQ) or 5-carboxymethylaminomethyl-2-geranylthiouridine (cmnm^5^ges^2^U).^2^ Sometimes these modifications appear to be permanent additions to the RNA, whereas in other cases, modifications like N^6^-methyladenosine (m^6^A) or 4-thiouridine (s4U) are reversible and highly regulated.^3,4^ Importantly, a single RNA molecule can harbor multiple modifications, resulting in numerous structures and functionalities. The implication here is that one gene (DNA sequence) can encode for myriad variations of mature RNA molecules based on these chemical decorations, the collection of which is termed the epitranscriptome. It is now appreciated that there is not one epitranscriptome as there is one genome, but instead a collection of epitranscriptomes depending on the cell type, environmental growth conditions, lifecycle stage, etc.^1^ tRNAs harbor the largest number of modifications in both prokaryotes and eukaryotes with roughly 14 per molecule on average;^5^ however, rRNA also require abundant modification for proper function. In eukaryotes, modifications are also regularly observed on mRNA and noncoding RNAs, whereas these modifications appear to be less common on the nonstructural prokaryotic RNAs.^5^ We definitely have a long way to go to completely understand all of the RNA modifications within even the simplest of organisms, but the NASEM report encourages the first steps toward defining a single epitranscriptome in full and the long-term goal of being able to even assess more complex and dynamic epitranscriptomes.

**Figure 1:**
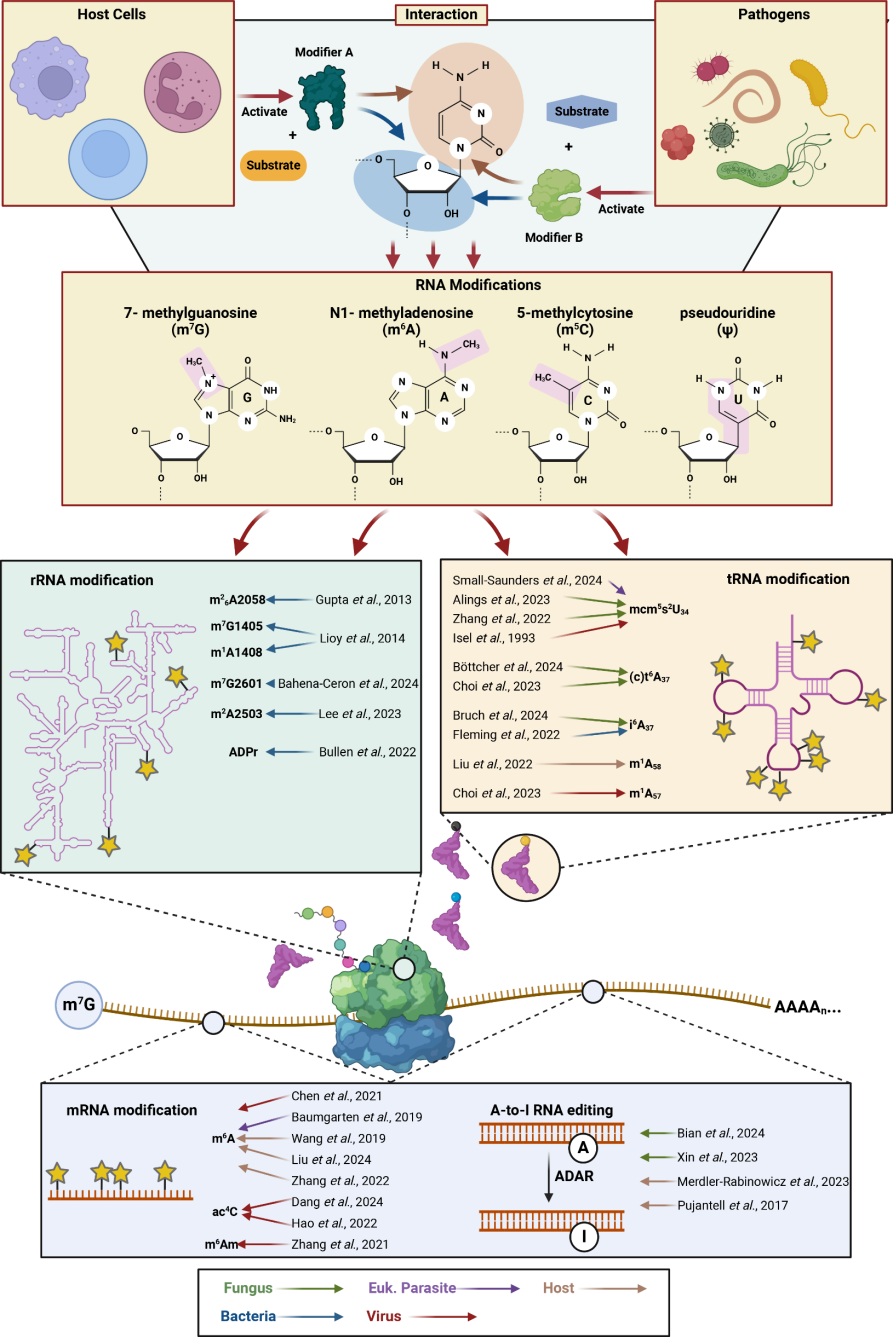
RNA modifications mediate microbial pathogenesis and host response. Despite an abundance of research into RNA modifications and microbial pathogenesis, we still know far too little about the importance of RNA modifications for the outcome of the host–pathogen interactions. The chemical structures of several well-described RNA modifications are highlighted (top).^9^ Several examples are given indicating common themes in RNA modification regulation as it relates to microbial pathogenesis and the host response to infection by viruses, bacteria, eukaryotic parasites, and fungi (bottom). Created in BioRender. [Blango, M. (2024) BioRender.com/b52i122].

**Figure 2:**
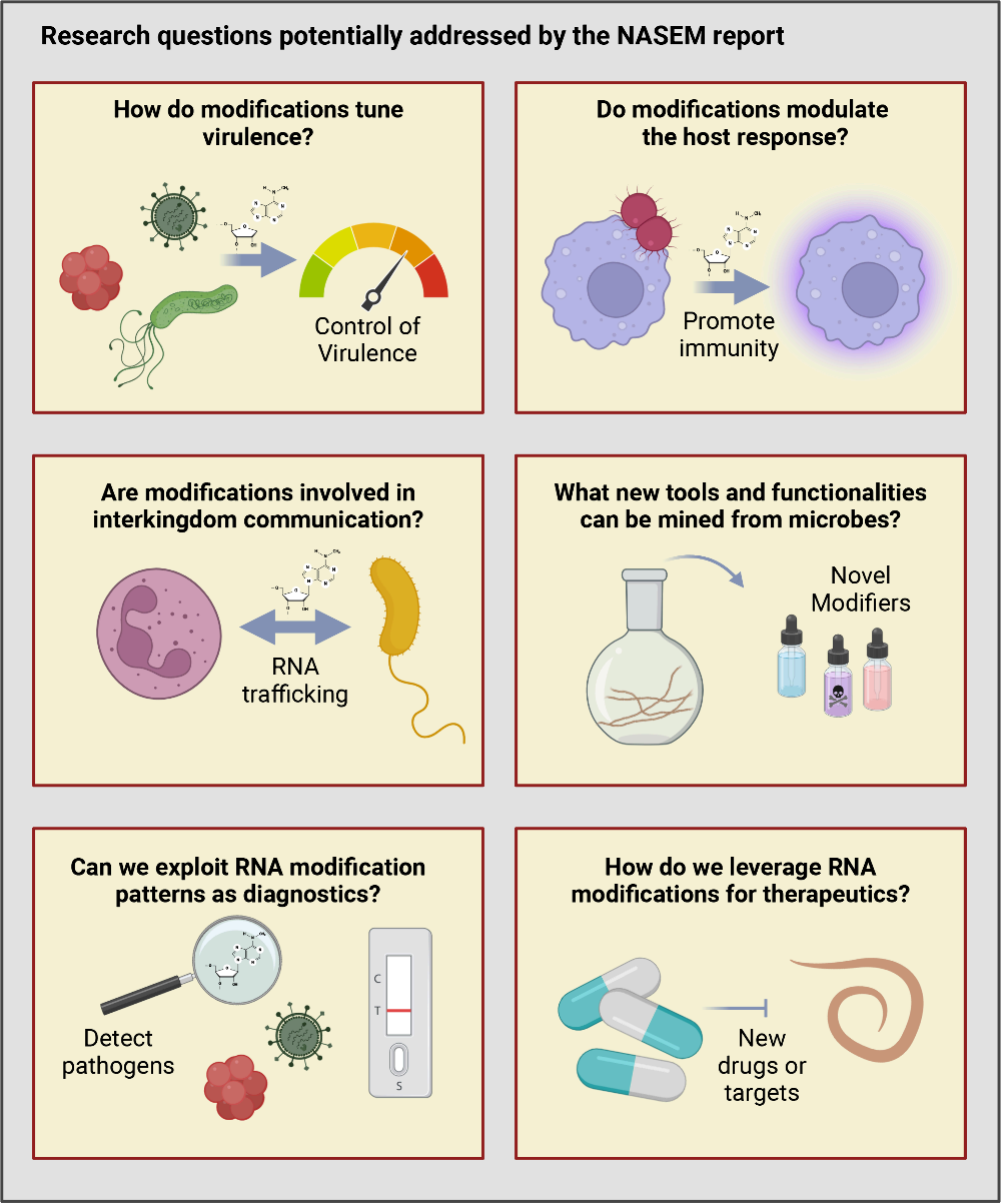
The future of RNA modifications in infectious disease research. A schematic showing where the study of RNA modifications may take us in terms of understanding pathogens and infectious disease, including RNA therapeutics. Created in BioRender. [Blango, M. (2024) BioRender.com/n17b280].

RNA modifications can currently be incompletely mapped using a variety of techniques, including mass spectrometry, next-generation RNA-sequencing technologies, and the emerging direct RNA sequencing approaches like the Oxford Nanopore Technologies (ONT) sequencing platform, among many others (Figure 1).^6^ Each of these techniques has strengths and weaknesses, but none are presently able to sequence all RNA modifications on a single RNA to determine the full epitranscriptome, a point firmly made by the NASEM report.^1^ The developing ONT sequencing approaches harbor a lot of promise for a future of mapping full epitranscriptomes, but as with any field in the early days, experts are still learning of the biases in the technology and improving de novo modification identification. Single-cell RNA-seq (scRNA-seq) is another approach that is pushing the boundaries of sensitivity and with great potential to improve our ability to assess all RNA modifications on all RNA of one individual cell. Approaches like these are currently being applied widely to the mapping of RNA modifications in the context of diverse human pathogens, including viruses, bacteria, fungi, and even archaea.^5,7,8^ In the remainder of this perspective, we will introduce some of the more compelling examples of the importance of RNA modifications in host–pathogenesis from the last few decades (Figure 1) and posit where the future of RNA modifications research may take us in combating infectious disease (Figure 2).

## Microbial Gene Regulation Is Modulated by RNA Modifications

Microbes have long been a source of pivotal research in RNA modifications, for example the study of *Saccharomyces cerevisiae* led to the first report of an RNA modification, pseudouridine, in 1957.^10^ Foundational work followed in abundance in many organisms, including bacteria like *Salmonella enterica*, both a human pathogen and laboratory workhorse.^11^ Studies of RNA modifications in *Salmonella* led to identification of a link between RNA modification and *his*(12) and *leu*(13) operon regulation and even supported the idea of flexibility within the genetic code.^14^ The field has expanded rapidly since then in many directions, and despite its origins in microbes, myriad intriguing questions remain to be answered surrounding the influence of modifications on pathogenesis, drug resistance, and stress response. The literature already supports the hypothesis that RNA modifications play essential roles in each of these processes, and many more (Table 1). In this article, we will only scratch the surface and demonstrate the amazing potential of RNA modifications in regulation of gene expression. We focus primarily on human pathogens, drawing also from studies performed using cell culture systems and mouse models in later sections. First, we will look at the pathogens themselves, as much work has already been done to understand how microbes control stress response and virulence pathways via RNA modifications.

**Table 1:** Influence of RNA Modifications on Pathogen Stress Resistance and Virulence: Selected Studies of Modifications in Human Pathogens^a^

modification	RNA	role(s) in pathogens	pathogen	ref
*Bacterial*				
ms^2^i^6^A	tRNA	Virulence and stress response controlled by modification of select tRNAs at A_37_	*E. coli* (UPEC)	(24)
cmnm^5^U	tRNA	Pathogenicity controlled by modification of select tRNAs at U_34_	*P. aeruginosa*	(28)
m^2^A	rRNA, tRNA	Reactive oxygen sensing and control of virulence	*E. faecalis*	(35)
Dimethylation	rRNA	Dimethylation of 23S A_2058_ promotes altered translation and resistance to erythromycin	*S. aureus*	(30)
m^7^G	rRNA	Confer aminoglycoside resistance	*E. coli*	(95)
m^7^G	rRNA	Methylation of the 23S at G_2601_ modulates virulence, growth, and biofilm formation	*S. aureus*	(33)
s^2^U	tRNA	Control of intracellular growth	*M. tuberculosis*	(26)
Queuosine	tRNA	LPS production	*E. coli* (ST131)	(27)
ADP-ribose	tRNA, rRNA	Bacterial RhsP2 functions as RNA-modifying effector protein to disrupt host structured noncoding RNAs	*P. aeruginosa*	(90)
Multiple	rRNA, tRNA	Numerous modification enzymes control resistance and susceptibility to a broad range of drugs	*V. cholerae*	(29)
*Fungal*				
i^6^A	tRNA	Stress response and drug resistance influenced by modification of A_37_	*A. fumigatus*	(21)
mcm^5^s^2^U	tRNA	In vivo virulence via modification of select tRNAs at U_34_	*S. cerevisiae*	(17)
			*C. albicans*	
t^6^A	tRNA	Influences adhesion and invasion	*C. albicans*	(18)
t^6^A	tRNA	Regulation of virulence factor production	*C. neoformans*	(96)
A-to-I	mRNA	A-to-I editing contributes to the sexual cycle	*F. graminearum*	(97, 98)
Multiple	tRNA	tRNA modifications are stable in response to ionizing radiation	*C. neoformans*	(99)
*Viral*				
mcm^5^s^2^U	tRNA	Modulate viral reverse transcription	HIV-1	(100)
m^6^A	Viral RNA	Maintains viral RNA stability and translation	HIV-1	(101)
m^6^A	Viral RNA	ALKBH5 coordinates viral and cellular response to low oxygen	Hepatitis B Virus	(102)
m^6^A	Viral sRNA	Modification of viral-encoded small RNA promotes replication	Parvovirus	(103)
m^6^A	Viral RNA	Modification promotes virus evasion of innate immune sensing	HIV-1	(104)
m^1^A	tRNA	Virus elicits modification removal to promote tRNA fragment production	RSV	(105)
2′-O-Me	Viral RNA	Internal modifications of viral genome promote escape	HIV-1	(106)
m^5^C	Viral RNA	Necessary for Aly/REF export factor recognition to promote mRNA export and translation	Hepatitis B Virus	(107)
*Parasites*				
mcm^5^s^2^U	tRNA	Regulation of s^2^U impacts artemisinin resistance	*P. falciparum*	(108)
m^6^A	mRNA	Altered m^6^A levels over asexual growth cycle	*P. falciparum*	(38)
Queuosine	tRNA	Promotes oxidative stress response and represses virulence	*E. histolytica*	(39)

a Abbreviations: ms^2^i^6^A_37_, 2-methylthio-*N*^6^-isopentenyladenosine 37; i^6^A_37_, *N*^6^-isopentenyladenosine 37; m^6^A, *N*^6^-methyladenosine; mcm^5^s^2^U_34_, 5-methoxycarbonylme-thyl-2-thiouridine 34; cmnm^5^U_34_, 5-carboxymethylaminomethyluridine 34; m^2^A, *N*^2^-methyladenosine; m^7^G, *N*^7^-methylguanosine; s^2^U, 2-thiouridine; t^6^A, *N*^6^-threonylcarbamoyladenosine; A-to-I, adenosine to inosine; m^6^A, *N*^6^-methyladenosine; 2′-O-Me, 2′-*O*-methylation

## tRNA Modifications in Pathogens Control Stress Response and Virulence

A lot of RNA modification research of pathogens to date has occurred in the context of tRNA modifications and their regulation of translational capacity in comparison to model bacteria (e.g., *Escherichia coli*) and fungi (e.g., *Saccharomyces cerevisiae*). Although powerhouses of genetics, laboratory strains of these organisms are often not particularly robust in terms of stress response.^15^ It is therefore not surprising that further investigation of core, conserved RNA modification enzymes in nonmodel organisms has already revealed previously unappreciated functionalities.

An important concept in the study of tRNA modifications is that of the modification tunable transcripts (MoTTs), which are transcripts with specific/biased codon usage whose translation can be modulated by specific tRNA modification levels.^16^ Alteration of tRNA modifications can thus lead to fine-tuning of the proteome via adjustment of the levels of tRNA modification enzymes or even the precursor metabolites required for modification. MoTTs provide the organism with an additional layer of regulatory control that coupled with the central importance of tRNA in gene expression gives prominence to modifications of the tRNA. This regulation manifests in many ways. For example, in a clinical isolate of the fungus *S. cerevisiae*, the Ncs2 enzyme required for 2-thiolation of tRNA was shown to harbor a single point mutation (Ncs2*) that facilitated growth at higher temperature and increased stress response and virulence compared to laboratory yeast strains.^17^ The authors determined that the point mutation led to increased 2-thiolation in the pathogenic strain at 37 °C, linking RNA modification and optimal translation firmly to virulence capacity in this case. A similar phenotype was observed in the opportunistic pathogenic yeast and frequent commensal *Candida albicans* for the Ncs2 ortholog; however, these functions are not always conserved. Another tRNA modification enzyme, Hma1, facilitated two distinguishable phenotypes through its threonylcarbamoyladenosine (t^6^A) dehydratase activity in the closely related *Candida* species, *C. albicans* and *C. dubliniensis*.^18^ This suggests that RNA modifications can be co-opted and evolved to fine-tune regulatory networks differently in each organism, hinting that we are likely to find a lot of new biology by broadening our search for RNA modification mechanisms.

In *Aspergillus fumigatus*, a filamentous fungal pathogen capable of causing infections ranging from allergy to deadly invasive aspergillosis, depletion of a particular tRNA modification via deletion of the catalytic subunit of the elongator complex resulted in numerous growth and stress phenotypes. Elongator is a conserved protein complex responsible for the 5-methoxycarbonylmethyl-2-thiouridine (mcm^5^s^2^U) modification of tRNA wobble uridine (U_34_) in a subset of tRNA isoacceptors. Modification of this position is typically necessary for efficient mRNA decoding and proper translation of proteins. In *A. fumigatus* the phenotypes associated with loss of this modification could be rescued by deletion of a transcription factor, CpcA, which is a yeast Gcn4 ortholog known to serve as an important sensor of altered translation efficiency caused by hypomodified tRNAs.^19^ This result linking the modification to control of metabolism differs to some degree from the conventional knowledge in yeast (and some higher eukaryotes), where elongator deletions result in phenotypes that cannot be rescued by Gcn4 deletion, e.g., proteotoxic stress and altered translation of mRNAs with higher ratios of target codon abundance, akin to MoTTs introduced above.^20^ The differences observed here suggest that even highly conserved pathways of modification can be leveraged for alternative roles in different organisms.

The concept of alternative functionalities between organisms continues with the isopentenyltransferase, Mod5, of *A. fumigatus* that modifies A_37_ of select tRNAs. Deletion of *mod5* results in increased resistance to the antifungal drug flucytosine, opposite to phenotypes observed in model fungi.^21^ Orthologs of Mod5 in bacteria, e.g., MiaA, are also linked to drug sensitivity^22^ and contribute to *vir* gene expression in the plant pathogen *Agrobacterium tumefaciens*,^23^*leu* operon expression in *Salmonella*,^13^ and stress response and virulence of extraintestinal pathogenic *E. coli* (ExPEC),^24^ adding a layer of importance atop a variety of phenotypes observed upon deletion in K12 laboratory *E. coli* strains.^25^ From these examples, it should not be surprising then that many tRNA modifications contribute to optimal virulence and stress response. For example, RNA modifications are linked to intracellular survival within the host for the devastating human pathogenic bacteria *Mycobacterium tuberculosis*, where MnmA-dependent tRNA uridine sulfation (s^2^U) was required for optimal intracellular growth.^26^ In *E. coli* ST131, the predominant ExPEC lineage worldwide, queuosine modification of tRNAs by QueF was tied to lipopolysaccharide production,^27^ and the GidA protein of the opportunistic bacterial pathogen *Pseudomonas aeruginosa* was shown to introduce a carboxymethylaminomethyl modification in selected tRNAs that modulates a switch between a pathogenic and general growth state.^28^ This switch was mediated through control of translation of virulence regulators by facilitating readthrough of rare codons requiring modification of wobble base 34 in select tRNAs. Such small changes leading to altered translational profiles highlights the rapidity and breadth of impact of even a single tRNA modification in the response of microbes to their environment.

Applying similar principles, a recent study of the role of rRNA and tRNA modifications on drug resistance in *Vibrio cholera* intriguingly uncovered unanticipated roles for many RNA modification enzymes in control of resistance or susceptibility to aminoglycosides, fluoroquinolones, β-lactams, chloramphenicol, and trimethoprim.^29^ A better understanding of the mechanisms of drug resistance for the causative agent of cholera has obvious potential to influence the way we treat *V. cholera* infections in the future. Collectively, these studies indicate an importance for tRNA modifications generally, but also demonstrate the potential of tRNA modifications of particular tRNA isoacceptors to serve as regulatory nodes capable of responding to stressful situations. Despite these important phenotypes, only a handful of tRNA modifications have been studied in depth in human pathogens, making this field ripe for the harvest of new regulatory mechanisms and impactful biology.

## rRNA Modifications Provide Regulatory Flexibility to Highly Conserved Molecules

Ribosomal RNA, like tRNA, is also heavily modified with important biological implications. Multiple studies have now documented the role of rRNA modifications in controlling diverse cellular responses relevant to virulence and stress resistance. For example, the erythromycin resistance methyltransferases (ERMs) of the Gram-positive bacterial pathogen *Staphylococcus aureus* promote resistance to the antibiotic erythromycin via dimethylation of a nucleotide in the large ribosomal subunit. Modification of the rRNA results in altered translation and a varied proteome.^30^ Interestingly, m^6^A_2058_-modified ribosomes of *S. aureus* are outcompeted by unmodified ribosomes, but during antibiotic treatment with macrolide, lincosamide, and streptogramin B antibiotics this phenotype is reversed, and the modified ribosomes give the bacteria an advantage.^31^ In fact, rRNA modification is a common way to modulate susceptibility to antibiotics, as shown repeatedly with the Cfr radical SAM enzyme that methylates A_2503_ (m^8^A_2503_) of *E. coli* 23S rRNA.^32^ Modifications of rRNA also impact virulence related phenotypes. Modification by RlmQ of *S. aureus* m^7^G_2601_ of the 23S rRNA impacts growth, virulence, and biofilm formation via modulation of the tRNA accommodation channel;^33^ the *rsmI* and *rsmH* genes of *S. aureus* were determined to be virulence genes responsible for 2′-*O*- and *N*^4^-methylations (m^4^*Cm*_1412_) of 16S rRNA;^34^ and the *Enterococcus faecalis*-modifying enzyme RlmN was recently shown to selectively alter modification upon direct sensing of reactive oxygen species (ROS).^35^ There, ROS, or sublethal doses of antibiotics capable of inducing ROS, led to decreases in N^2^-methyladenosine (m^2^A) for both the 23S rRNA and tRNAs, with implications for an environmentally responsive, dynamic RNA modification system. Similarly, approaches using direct nanopore sequencing have confirmed the modification patterns of rRNA to be complex and dynamic under stress just like those of tRNA,^36^ spotlighting again the importance of mapping the epitranscriptome under many different conditions as proposed in the NASEM report.^1^

## Environmental and Physiological Adaptations of Microbes Are Fueled by RNA Modifications

A variety of RNA modifications have been studied in the context of parasitic protozoan biology,^37^ with many of the same themes emerging as occur in the fungi or even higher eukaryotes. For example, the asexual life cycle of the unicellular protozoan parasite *Plasmodium falciparum* relies on dynamic amounts of m^6^A methylation to fine-tune gene expression via modulation of RNA stability and translational efficiency across the lifecycle.^38^ Another example linking environment to pathogenesis comes from the anaerobic parasitic amoebozoan *Entamoeba histolytica*, where the hypermodified nucleobase queuine modulates oxidative stress response and serves as a virulence attenuator.^39^ Queuine, which must be taken up from the environment by eukaryotes, is incorporated into tRNA by a tRNA-guanine transglycosylase (EhTGT) in *E. histolytica*, resulting in a queuosine-modified ribonucleoside in a small subset of tRNAs. An abundance of queuine represses virulence by downregulating expression of virulence-associated genes. This case highlights the importance of the environment and nutritional availability/supplementation on modification status and the conclusions we draw about modification patterns in controlled laboratory settings. Surely, much more can be learned from studying modifications in unconventional, albeit challenging systems like the protozoan parasites.

It is well-documented that RNA modifications play essential roles in viral pathogenesis.^40−42^ They contribute to viral RNA stability^43^ as well as mRNA capping to limit detection by host pattern recognition receptors, e.g., by encoding their own 2′-O-methyltransferase (2′-O-MTase) for cap addition.^44,45^ Modifications are also often required for full viral functionality, as observed with cytidine methylation of the pregenomic RNA required for a proper hepatitis B virus life cycle.^46^ Similarly, m^6^A modification of the human immunodeficiency virus (HIV) viral RNA mediates increased stability through binding of the host m^6^A reader protein YTHDF2.^47^ In this case, the virus relies on modification to exploit a host protein to stabilize its genome. In another variation of this theme, it was recently postulated that the human ‘apolipoprotein B mRNA-editing enzyme, catalytic polypeptide-like’ (APOBEC) family proteins contribute to SARS-CoV-2 evolution directly in the host by introducing cytosine-to-uracil (C-to-U) transitions in the viral genome.^48^ The overall mechanism is unclear, but the observed overrepresentation of C-to-U transitions suggests that host RNA modification pathways are likely being co-opted to facilitate viral pathogenesis and evolution at least in some cases. The role of RNA modifications in the host response, particularly regarding control of viral replication, is an expansive topic on its own, which will be addressed in more detail in the following sections.

## Regulation of mRNA Methylation in Higher Eukaryotes Controls Host Response

The host response to infection is complex, relying on both broad general responses and specific actions against individual pathogens; all of which must be coordinated and, in many cases, recalcitrant to attack by the pathogen itself. The mechanistic role of RNA modifications in immunity remains poorly explored, but it is already obvious that modifications play a significant role in regulating the immune response to a wide variety of pathogens by influencing RNA structure and stability or interaction with RNA binding proteins (Table 2).^49,50^

**Table 2:** Influence of RNA Modifications on Host Response to Infection: Selected Studies of RNA Modifications Involved in the Host Response to Infection^a^

modification	RNA	role(s) in host	host	ref
m^1^A	tRNA	Improve MYC translation to promote T cell expansion	Mouse	(109)
m^6^A	mRNA	hnRNPA2B1 facilitates modification and trafficking of *CGAS*, *IFI16*, and *STING* mRNAs	Human/Mouse	(110)
m^6^A	mRNA	Modifications are dynamically regulated across infection	Mouse	(53, 54)
m^6^A	mRNA	Negative regulator of immunity in CMV infection	Human/Mouse	(55)
m^6^A	mRNA	Promote granulopoiesis and neutrophil mobilization	Human/Mouse	(59)
m^6^A	mRNA	Demethylation results in antiviral transcripts trapped in nucleus	Mouse	(58)
m^6^A	mRNA	Modification of α-ketoglutarate dehydrogenase (*OGDH*) mRNA reduces stability and protein expression to limit viral replication	Human/Mouse	(57)
m^6^A	mRNA	Inhibition of hepatitis B virus protein expression	Human	(111)
m^6^Am	mRNA	HIV viral protein R (Vpr) interacts with PCIF1 methyltransferase to facilitate ubiquitination and degradation preventing m^6^A adjacent to m^7^G cap	Human	(112)
NAD	snRNA/snoRNA	snRNA and snoRNA lost NAD+ cap when infected with HIV-1	Human	(113)
m^5^C	mRNA	m^5^C of IFR3 mRNA negatively regulates IFN I responses during viral infections	Human	(114)
m^5^C	lncRNA	Depletion of NSUN2 (m^5^C methyltransferase) leads to increased interferon I response and viral suppression	Human/Mouse	(115)
A-to-I	dsRNA	A-to-I editing of viral RNA restricts infection	Human/Mouse	(63−65)
A-to-I	mRNA	A-to-I editing varied from tissues and cell types during a variety of infections	Human/Mouse	(70−73, 116)
A-to-I	dsRNA	ADAR1 regulates immune functions	Human	(117)
A-to-I	Viral dsRNA	ADAR promotes viral evolution	Human	(67, 68)
ac^4^C	mRNA	Control of alphavirus and enterovirus 71	Human/African Green Monkey	(79, 80)
Ψ	mRNA, ncRNA	Pseudouridine is added to host and HIV-1 viral transcripts	Human	(88)
C-to-U	mRNA	C-to-U editing by APOBEC3A restricts viral infection	Human	(75, 76)
Glycosylation	exRNA	Control of neutrophil recruitment	Mouse	(118)
Multiple	Total RNA	*Toxoplasma gondii* infection changes modification patterns in mouse spleen and liver	Mouse	(119)

a Abbreviations: m^6^A, *N*^6^-methyladenosine; m^6^Am, *N*^6^,2′-*O*-dimethyladenosine; NAD, nicotinamide adenine dinucleotide; m^5^C, 5-methylcytosine; A-to-I, adenosine to inosine; ac^4^C, *N*^4^-acetylcytidine; Ψ, pseudouridine; C-to-U, cytosine to uracil

One of the most abundant modifications in higher eukaryotes is methylation of the N^6^ position of adenosine, denoted N^6^-methyladenosine (m^6^A).^51^ This modification contributes expansively to gene regulation, including during immunity.^51,52^ The “writer”, “reader”, and “eraser” proteins of m^6^A metabolism that add, interact with, or remove m^6^A use this modification to coordinate multiple aspects of the immune response, including immune cell differentiation, proliferation, activation, and even polarization, among others.^52^ The coordinated rewriting of m^6^A during infection has been linked to viral repression in the case of the murine pathogen vesicular stomatitis virus (VSV) and binding of reader proteins to m^6^A-modified transcripts is linked to control of antiviral defense against murine cytomegalovirus (CMV)^53^ reminiscent of the example described above for HIV.^47^ Fungal pathogens are capable of influencing host m^6^A RNA modification patterns during infection as well. For example, m^6^A levels appear to increase throughout the course of infection in the eye during *Fusarium solani* infection.^54^ So far, the implications of large-scale rewriting of m^6^A modifications in host defense are mostly descriptive, but as techniques and sensitivities improve, we will likely gain a better view of the complexities of modifications during infection. This is just one area where the advances gained in line with the NASEM report will have concrete effects on the ability to better interrogate RNA modifications during infection.

The m^6^A modification can also serve as a negative regulator of immunity, as occurs during human CMV infection, where m^6^A modification is relied upon to repress the interferon response and maintain cellular homeostasis.^55^ A similar outcome is observed for the m^6^A reader YTHDF3, which regulates translation of the important transcriptional regulator FOXO3 independently of its m^6^A activity to selectively inhibit interferon (IFN)-stimulated gene expression.^56^ In this case, the RNA modification machinery has been repurposed for an additional modification-independent regulation, underscoring the dangers of assigning functions for modifications based only on purported protein activities. The immune response is a highly complex regulatory network that requires numerous inputs and adjustments for proper function. Although limited, the examples described here hint that this network is commonly fine-tuned or even drastically altered by the presence, absence, or dynamics of RNA modifications during infection. In the future, higher resolution mapping of RNA modifications during the host response should allow us to better appreciate the influence of modification dynamics on host response.

As with any complex biological system, multiple pathways can lead to the same outcome. For example, a host may add an RNA modification like m^6^A directly using a writer enzyme or alternatively control the removal of RNA modifications by eraser enzymes to influence their response and maintain a modification, as occurs in the mouse host during vesicular stomatitis virus (VSV) infection. Here, the murine host inhibits the activity of the RNA m^6^A demethylase ALKBH5 to limit viral replication by restricting production of itaconate through destabilization of the α-ketoglutarate dehydrogenase (*OGDH*) mRNA.^57^ In parallel, ALKBH5 itself can be recruited by the DEAD-box (DDX) RNA helicase DDX46 to demethylate m^6^A-modified antiviral transcripts and trap them in the nucleus limiting innate immunity during VSV infection.^58^ Interestingly, during bacterial infection ALKBH5 performs a different function and promotes granulopoiesis and neutrophil mobilization during cecal ligation-induced polymicrobial sepsis.^59^ Collectively these studies reveal the dynamic networks of “writer”, “reader”, and “eraser” proteins required during different infection situations and show that varied strategies of regulation are used against different microbial challenges.

## Host Organisms Use RNA Modifications to Modulate the Response to Pathogens

Another system that is leveraged for a variety of cellular functions relies on Adenosine-to-Inosine (A-to-I) editing, which occurs widely in pre-mRNA, mature mRNA, and ncRNA. In mammals, this modification is broadly mediated by adenosine deaminase acting on RNA (ADAR) and more specifically on tRNA by adenosine deaminase acting on tRNA (ADAT) proteins. Most organisms harbor orthologs of the ADATs, important for installing inosine in the wobble position of some tRNAs, whereas only higher eukaryotes appear to have canonical ADAR proteins. The ADAR proteins have been linked to gene regulation of immunity in multiple systems,^60^ but the most extensive studies relevant to this perspective come from mouse and human studies.^61,62^ The specificity of ADAR proteins to bind and (hyper)edit long stretches of perfectly duplexed double-stranded RNA (dsRNA) leads to the obvious hypothesis that ADAR could modify viral dsRNA to restrict infection by scrambling the genome of RNA viruses; however, the story is much more complex. While in some cases direct modification of viral RNA does in fact seem inhibitory (e.g., measles virus),^63−65^ ADAR activity can sometimes facilitate infection^66^ or even promote viral evolution.^67,68^ These results suggest that the host response to viral infection can be both promoted and inhibited by the activity of ADAR proteins depending on the virus, cell type, and stage of infection.^69^ RNA editing patterns within the host also change in a pathogen-dependent manner, as observed now in numerous studies of viral infection^70−72^ but also intracellular bacterial infections.^73^ All these interactions must be considered in the context of the normal housekeeping functions of ADAR in marking host dsRNA as “self” to limit inflammation by dsRBP sensor proteins like MDA-5,^74^ complicating further the already heterogeneous RNA modification landscape during infection.

Finally, several other RNA modifications have been investigated to some detail in the host response to pathogens. For example, human APOBEC3A mediates C-to-U RNA editing as discussed above and functions like ADAR editing in regard to restriction of viral pathogens and coordination of the host response to infection.^75,76^ APOBEC3A is a member of a larger family of enzymes, many of which contain RNA editing activity. Certainly, more investigation of the contributions of these enzymes will reveal novel biology relevant to infection. Hints already imply that a complex interplay exists from studies of SARS-CoV-2.^77^ 5-methylcytosine (m^5^C), another modification of cytosine, has been linked to negative regulation of the interferon response, where it was shown that depletion of the NSUN2 m^5^C methyltransferase enhanced the interferon response and viral suppression.^78^ These results are consistent with additional work on H1N1 influenza A virus, where m^5^C was observed to be deposited on lncRNAs following infection of human A549 epithelial cells.^60^ Cytidine is also modified by N-acetyltransferases (ac^4^C) and again linked to control of viral infection. N-acetyltransferase 10 was shown to regulate replication of alphavirus^79^ and enterovirus 71,^80^ two viruses capable of causing severe central nervous system destruction. Improved mapping of modifications like ac^4^C and m^5^C will likely reveal additional mechanisms associated with viral pathogenesis and link this modification to other host-microbe interactions in the future.

## Technological Advances Have Improved Our Assessment of Modifications during Infection

Technology has advanced rapidly around RNA modifications, and efforts have been made to map a variety of modifications during the host response as discussed throughout this perspective. Advances in RNA-seq technology have facilitated the large-scale identification of specific modifications in a variety of pathogens (Table 1), albeit with moderate levels of noise.^6,81^ Chemical- and antibody-assisted methods have provided orthogonal evidence and removed some bias from the initial direct sequencing approaches,^6^ and the latest generation of nanopore direct RNA sequencing has further refined our understanding.^21,82,83^ Emerging combinations of chemical ligation approaches^84,85^ with sequencing technology will likely prove better still, but are unlikely to facilitate the identification of all modifications on a single RNA in the near-term. The story is similar for mass spectrometry analysis, with new more sensitive devices constantly reaching the market and improved analysis techniques,^86,87^ we now have an unprecedented and frankly amazing view of many modifications. Our incomplete set of chemical standards remains a limitation, and in nearly all cases, many exciting questions remain. The mapping of RNA modifications on each and every RNA in a cell will facilitate the identification of novel biological mechanisms of regulation and improve our understanding of the host response to microbial challenge but will also force us to ask more questions and evolve our thinking around RNA modifications and infection. For example, a recent study mapping pseudouridine on ncRNA and mRNA during HIV-1 infection observed that the enzyme required for pseudouridylation of mRNA in humans remains surprisingly poorly understood, highlighting the fact that we still have only a rudimentary knowledge of many of the mechanisms behind modifications, despite all these technological advances.^88^ In the next section, we will explore a few more unanswered questions that could be answered by efforts stemming from the NASEM report.

## Outstanding Questions: What Do We Stand to Gain from Mapping All RNA Modifications?

As seen in the previous sections, RNA modifications are clearly playing important, impactful roles in regulation of host pathogen interactions, but the elucidation of all RNA modifications within a particular epitranscriptome is likely to be further transformative in numerous ways. We expect that such an RNA modification moonshot will result in **1)** an improved mechanistic understanding of pathogenesis at the molecular level and reveal novel players in infection biology, **2)** facilitate new and more accessible technology at lower cost, and **3)** promote realization of new therapeutics and treatment strategies (Figure 2). As highlighted in the NASEM report, such strategic investments in science are not only a boon to research but also provide a quantifiable return on investment to the benefit of society.

The first benefit of defining RNA modifications in their entirety will be a more mechanistic understanding of host–pathogenesis. The interaction of host and pathogen is frequently described as an evolutionary molecular arms race to gain the advantage over the opposing organism. The role of RNA modifications in shaping these interactions and facilitating flexibility beyond that encoded in the genome remains poorly investigated, but certainly a compelling question. Examples mentioned here do support the ability of RNA modifications to facilitate viral evolution,^67,68^ but how these modifications may prime the host for more rapid evolution remains poorly understood.^89^ Relatedly, there is likely much to learn about the evolution of RNA modification distribution across single RNA molecules in hosts and pathogens, which could potentially be revealed by the biological weaknesses exploited by each opposing partner during interaction. Another area where intensified interest in RNA modification biology may uncover new regulation is surrounding pathogen effectors, which are best described in bacteria and plant fungal/oomycete pathogens as secreted proteins involved in modulating or manipulating host responses. Currently, limited examples exist of RNA modification enzymes capable of serving as effectors to impart a cross-kingdom RNA modification (e.g., bacterial or fungal enzymes secreted to modify human RNA),^90^ but it seems likely that many more instances will be discovered with time. We are particularly intrigued by examples from human fungal pathogens, where research typically lags that of viral and bacterial pathogens. Advances in de novo mapping of RNA modifications will likely prove pivotal in defining these rare modifications introduced by pathogens and that may only appear in conjunction with known, well-characterized modifications or at low frequency in dying cells during infection. This is an exciting area that could benefit tremendously from improved modification mapping sensitivities.

The NASEM report mentions on several occasions the importance of mining new enzymatic activities from microbes to advance the technology required to map RNA modifications. The elucidation of novel microbial factors that can be leveraged for RNA modification research will in turn reveal new information about the microbes themselves. These advancements in technology in parallel with improved computing capacity will certainly supercharge research efforts into RNA modifications during infection. Currently, investigating RNA modifications during the host pathogen interaction is challenging due to limited biological materials and complicated samples due to nucleic acid contributions from multiple partners. Increased sensitivity and versatility of methods will hopefully improve the chances of success in this area. Challenges will remain. How can we assign modifications to the host when we do not know the complete modification repertoire of the pathogen? We will also need solutions to mapping modifications in complex disease states and polymicrobial communities, where all the microbial players may not even be yet known.

The goal of studying pathogens and host–pathogenesis is to inform better strategies for treatment of these invaders in the clinic. RNA modification research has already proven to be valuable in this regard by demonstrating an importance in influencing drug resistance, modulating pathogenesis, and facilitating new biotechnology, e.g., the mRNA vaccines that ultimately limited further SARS-CoV-2 spread. More generally, the study of RNA in the context of RNA-based therapeutics is already changing the way we consider treatment of infectious disease,^91,92^ with new vaccines in the pipeline and RNA-based drugs to fight viral infections in development. The NASEM report provides a roadmap, but how this plan will be used and adapted by individual subfields will be an additional challenge, particularly when ensuring that the technological gains are shared openly and widely in a reproducible manner.

The list of outstanding questions in infectious disease research that may be answerable with a newfound capability to map full epitranscriptomes is enormous, but we definitely had a few personal favorites in brainstorming for this perspective. First, we are excited to learn how RNA modification patterns influence RNA trafficking, particularly in the context of RNA secretion in association with extracellular vesicles. The primary advances in this area may not initially come from the study of host pathogenesis,^93,94^ but it seems likely that understanding how chemical changes to RNA influences their intracellular, intercellular, or even interkingdom trafficking will be of major importance in therapeutically delivering RNA to specific niches in the future. We are also excited to see studies of the full epitranscriptome from individual cell types during infection. Just as mRNA isoforms differ widely between cell types, we expect that RNA modification patterns will follow suit, particularly in response to different pathogens. Coalescing all this information into usable models and tractable databases will be a huge challenge for the future, likely requiring clever computational solutions as mentioned by the NASEM report. Finally, how will mapping of RNA modifications change diagnostics? It seems possible that in the future, rapid mapping of cellular RNA modifications from a host blood or urine sample could be used together with deep learning to identify all sorts of maladies, including genetic disorders, cancers, or even pathogens typically hidden below detection limits.

## Conclusions

The future of RNA modifications research holds amazing potential to understand the molecular world. As the human genome project completely changed our research landscape, so too will a major investment in unraveling the mysteries of RNA modifications. As in many cases, studies of host–pathogenesis are likely to mostly follow major breakthroughs from more tractable systems, but as in the past, investigation of host–pathogen interactions will also reveal features of the molecular world that may be concealed in these same tractable model systems: likely we will see entirely new functions for RNA and RNA modifications emerge from inspection of pathogenic microbes. Major investment in strategic initiatives tends to simultaneously improve accessibility to previously considered “specialist” assays and make more commonplace technically complex equipment, while also bolstering availability and lowering prices of rare reagents. If the NASEM report is followed even partially toward completion, we anticipate a future alight with our understanding of the role of RNA in microbial pathogenesis and host immunity.
